# Immune mechanism of IgA vasculitis with nephritis: a narrative review

**DOI:** 10.3389/fimmu.2026.1754289

**Published:** 2026-07-31

**Authors:** Chan Xiu, Jun Zhang, Baozhao Ju, Tao Ma, Jing Lv

**Affiliations:** 1 Affiliated Hospital of Liaoning University of Traditional Chinese Medicine, Shenyang, China; 2 Liaoning University of Traditional Chinese Medicine, Shenyang, China; 3 Shenyang Orthopedic Hospital, Shenyang, China

**Keywords:** cellular immunity, cytokines, humoral immunity, IgA vasculitis, IgA vasculitis with nephritis, immune mechanism

## Abstract

IgA vasculitis with nephritis (IgAVN), also known as Henoch-Schönlein purpura nephritis (HSPN) is a kidney disease secondary to IgA vasculitis (IgAV). While most pediatric IgAVN patients have a favorable prognosis, a minority may develop renal insufficiency or even progress to end-stage renal disease, compromising quality of life and imposing a significant burden on families. The etiology of IgAVN is complex, involving intricate immune system dysregulation. Elucidating its pathogenesis is crucial for guiding treatment and improving patient outcomes. Therefore, this review comprehensively synthesizes the immunopathogenic mechanisms of IgAVN including humoral, cellular, and cytokine networks to provide a robust theoretical framework for the rational design of novel therapeutic agents.

## 1 Introduction

Immunoglobulin A vasculitis with nephritis (IgAVN), also known as Henoch-Schönlein purpura nephritis (HSPN), represents a severe complication of Immunoglobulin A vasculitis (IgAV), an IgA-mediated immune complex systemic small-vessel vasculitis. IgAVN primarily affects the kidneys, with its pathological process involving complex interactions among multiple immune cells and molecules (1). Deposition of IgA immune complexes in the glomerular mesangium induces localized inflammatory injury, ultimately leading to IgA nephropathy (IgAN), a kidney-specific glomerular disease. In contrast, accumulation of these complexes on the endothelium of small vessels in multiple systemic sites (e.g., skin, gastrointestinal tract, and renal microvasculature) activates the complement cascade and induces systemic vasculitis, manifesting as IgAV, a syndrome characterized by multisystem mucosal and vascular inflammatory responses. IgAVN shares overlapping immunopathological features with IgAN, but also shows distinct clinical and histopathological characteristics (2–4).

Global epidemiological studies indicate that IgAV is highly prevalent in Asia, with a peak incidence in children aged 4 to 6 years (5). Renal involvement (IgAVN) occurs in 20% to 80% of pediatric cases, and although often self-limiting, it remains a significant cause of chronic renal failure, particularly in adults (5–7). Although growing evidence suggests a more favorable prognosis for IgAVN compared to IgAN, some acute-phase cases develop life-threatening sequelae including acute kidney injury, gastrointestinal bleeding, and intestinal perforation, complications that jeopardize patient survival and impact long-term outcomes.

In recent years, advances in immunology and molecular biology techniques have expanded our understanding of the immunological mechanisms underlying IgAVN beyond simple IgA deposition to reveal complex dysregulation within the immune network. Research indicates that the pathogenesis of IgAVN involves the production of abnormally glycosylated IgA1 molecules, dysfunction of T and B cells, macrophage activation, and an inflammatory cascade involving multiple cytokines (8). Humoral abnormalities, most notably aberrant O-lycosylation of IgA1 with increased circulating galactose-deficient IgA1 (Gd-IgA1) and frequently elevated serum IgE, lead to the formation of pathogenic immune complexes that deposit in the glomerular mesangium and small-vessel endothelium and activate complement pathways. These humoral events initiate and are amplified by dysregulated cellular immunity including Th1/Th2 imbalance, Th17/Treg imbalance, expansion of follicular helper T (Tfh) cells and deficiency of regulatory B (Breg) cells which promotes sustained B cell activation, pathological IgA production and persistent inflammation. A network of inflammatory cytokines (TNF-α, IL-6, IL-16, IL-18, IL-17, IL-21, IL-29, IL-33) mediates endothelial activation, mesangial proliferation, epithelial-mesenchymal transition (EMT), extracellular matrix deposition and fibrosis, thereby determining disease severity and progression toward chronic renal impairment. Therefore, this review synthesizes and distills the immunopathogenic mechanisms of IgAVN based on existing literature, aiming to provide a robust theoretical framework for the rational design of novel therapeutic agents and the optimization of clinical management strategies for IgAVN.

## 2 Humoral immunity

Humoral immune abnormalities constitute a primary and upstream axis in the pathogenesis of IgAVN. Aberrant O-glycosylation of IgA1 resulting in Gd-IgA1 promotes the generation of anti-Gd-IgA1 autoantibodies and circulating immune complexes that can deposit in the glomerular mesangium and activate complement pathways. Concurrently, elevated serum IgE levels which commonly observed in IgAVN can sensitize mast cells and basophils through FcϵRI and contribute to type I hypersensitivity and systemic small-vessel vasculitis, which may increase vascular permeability and thereby facilitate immune complex access to renal compartments. This section will therefore focus on the humoral components (IgA, IgE) that act as initiators of downstream cellular responses and inflammatory cascades described in following section of cellular immunity and inflammatory cytokines. .

### 2.1 Glycosylated IgA1

**Figure 1:**
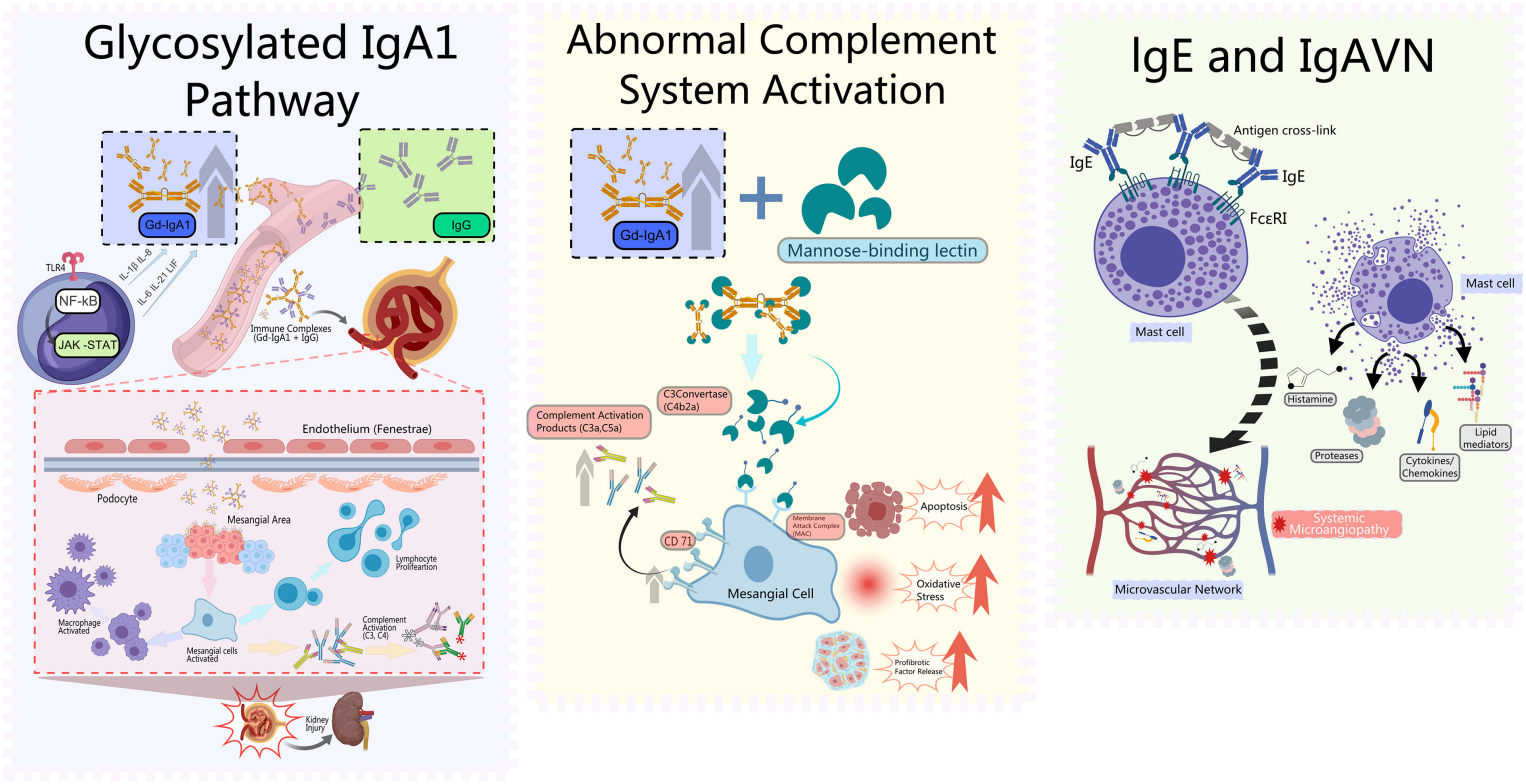
The core role of humoral immunity mechanisms in lgAVN: the production of abnormal glycosylated IgA1 (Gd-IgA1), which lead to immune complex formation and renal injury. Gd-IgA1, resulting from abnormal glycosylation, is associated with disease severity and triggers inflammation and complement activation. Additionally, elevated IgE levels are linked to IgAVN severity, as IgE promotes allergic inflammation and sensitizes mast cells, contributing to the disease’s progression. These mechanisms, including abnormal glycosylation, complement activation, and IgE involvement, underpin the immune complex-complement-inflammation cycle in IgAVN.

IgA has two subclasses, namely IgA1 and IgA2. Galactosodeficient IgA1 (Gd-IgA1) is the core pathogenic molecule in IgAN and IgAVN. The main forms include N-acetylgalactosamine (GalNAc) without sialic acid, galactose (Gal), and sialic acid. When the O-linked glycosylation site in the hinge region of IgA1 protein undergoes abnormal glycosylation, it fails to bind to Gal but instead binds to GalNAc (**Figure 1**). Consequently, its molecular structure undergoes structural alterations. Its molecular structure is characterized by the absence of galactose in the O-glycan of the hinge region. Multiple studies have demonstrated through mass spectrometry and lectin binding assays that both glomerular deposits and circulating IgA1 in IgAN patients are enriched with Gd-IgA1. Levels are significantly higher than in healthy controls and correlate positively with disease severity (9–11). Elevated Gd-IgA1 induces serum IgA and IgG autoantibodies, where IgG may form immune complexes with Gd-IgA1. These complexes traverse the glomerular endothelial fenestrations, deposit in the mesangial region, activate mesangial cells, and trigger macrophage and lymphocyte proliferation. Alternatively, they activate the complement pathway, initiating inflammatory responses that ultimately cause renal damage.

The production of Gd-IgA1 also involves cellular immunity, cytokines, and multiple signaling pathways. Abnormal regulation of the glycosyltransferase system within B cells is implicated in Gd-IgA1 production. The cytokine microenvironment critically regulates B-cell glycosylation processes. Pro-inflammatory cytokines such as interleukin (IL)-6, IL-21, and leukemia inhibitory factor (LIF) can upregulate Gd-IgA1 production via the JAK-STAT signaling pathway (12, 13). Toll-like receptor (TLR) signaling also participates in regulation. TLR4 indirectly influences IgA1 glycosylation by upregulating IL-1β and IL-8 expression through the NF-κB pathway (14).

### 2.2 Abnormal complement system activation

Abnormal complement system activation is a key component in IgA-associated nephropathy (IgAN) renal injury. Mannose-binding lectin (MBL) initiates the complement cascade by binding to the abnormal glycosylation of Gd-IgA1, generating C3 convertase (C4b2a). With the assistance of the alternative pathway, this further leads to the formation of the membrane attack complex (MAC, C5b-9) formation via the alternative pathway (15, 16), causing cell lysis or sub-lytic injury manifested as apoptosis, oxidative stress, and release of pro-fibrotic factors (17). Complement activation products (e.g., C3a, C5a) also upregulate mesangial cell CD71 expression, forming a positive feedback loop that exacerbates immune complex deposition. Together, these mechanisms constitute the vicious ‘immune complex-complement-inflammation’ cycle in IgAVN, providing a theoretical basis for targeted therapies (6) (**Figure 1**).

### 2.3 IgE

The role of IgE in IgAVN pathogenesis has garnered increasing attention. A study of 260 pediatric IgAV patients revealed significantly elevated IgE levels compared to healthy controls, with strong correlation to disease severity (18). IgE is associated with Type I hypersensitivity and allergic inflammation. Antigen-mediated cross-linking of FcϵRI-bound IgE triggers rapid release of multiple effector molecules, including histamine, proteases, cytokines, chemokines, and lipid mediators (19). Studies confirm that IgE sensitizes mast cells in human skin tissue, prompting target cells to release vasoactive substances that mediate systemic microangiopathy and promote IgAV pathogenesis (20).

In summary, humoral immune mechanisms in the blood play a pivotal role in IgAVN. Specifically, the production of abnormally glycosylated IgA1, immune complex deposition, and complement activation are inextricably linked to the pathogenesis of IgAVN, emerging as core drivers of the disease and potential therapeutic targets (**Figure 1**).

## 3 Cellular immunity

The cellular immune pathogenesis of IgAVN is characterized by the core features of functional polarization imbalance of multi-lineage T cell subsets and effector dysfunction of innate immune cells, including Th1/Th2 immune response skewing toward Th2-dominant polarization that induces allergy-prone inflammatory reactions, Th17/Treg ratio imbalance that mediates autoimmune injury effects, abnormal activation of Tfh that leads to dysregulated secretion of pathogenic polymeric IgA, as well as natural killer (NK) cell metabolic reprogramming disorders and macrophage M1/M2 polarization imbalance that disrupt the renal tissue injury-repair process. These multiple immunopathological links interact synergistically, and ultimately drive the occurrence, progression and renal function impairment of IgAVN through sustained activation of local renal immune-inflammatory cascade reactions.

### 3.1 Th1, Th2

Children with IgAVN exhibit disrupted T lymphocyte subsets and functional abnormalities. Studies indicate that both IgAV and IgAVN groups have significantly higher Th2 cell counts than healthy controls, while Th1 cell counts are markedly reduced (21). Th1 cells primarily participate in anti-intracellular pathogen infection and cellular immunity, secreting cytokines such as interferon-gamma (IFN-γ), tumor necrosis factor-alpha (TNF-α), and IL-2. Among these, IFN-γ is the hallmark cytokine of the Th1 subset. IFN-γ is a multifunctional immunoregulatory cytokine playing a central role in Th1-type immune responses. It drives cytokine expression and activates inflammatory cells, while also serving as a key immunomodulator regulating Th17 cell development and activity, neutrophil chemotaxis, and enhancing regulatory T cell (Treg) function (22). IL-2 promotes T cell and natural killer (NK) cell proliferation while enhancing T cell cytotoxic effects and NK cell activity. Th2 cells primarily participate in combating extracellular pathogen infections and humoral immunity, secreting inflammatory cytokines such as IL-3, IL-4, IL-5, IL-6, IL-10, and IL-13 (23). IFN-γ suppresses CD4^+^ T cell differentiation toward Th2 cells, while high concentrations of IL-4 inhibit CD4^+^ T cell differentiation toward Th1 cells (24). When Th1 function is impaired, the inhibitory effect on Th2 cells is lifted, leading to Th2 hyperfunction and increased susceptibility to allergic diseases. Extensive research indicates that pediatric IgAVN patients exhibit Th2 cell-dominant activation with a significantly imbalanced Th1/Th2 ratio, suggesting that Th1/Th2 imbalance plays a crucial role in the pathogenesis of IgAVN (25).

### 3.2 Th17, Treg

In children with IgAVN, the proportion of helper T cell 17 (Th17) in peripheral blood was significantly higher than that in normal controls during both the acute phase and remission phase, while the proportion of regulatory T cells (Treg) was significantly lower than that in normal controls (26). Th17 is a pro-inflammatory T cell subset that has been demonstrated to be a major inducer of autoimmunity, and its functional upregulation triggers tissue inflammation (26). Treg cells play a crucial role in maintaining tissue homeostasis and represent a protective T cell subset. The developmental pathways of Treg and Th17 cells are mutually regulated. Under inflammatory conditions, precursor Treg cells can be converted into Th17 cells, which promote the onset and progression of certain autoimmune diseases by expressing IL-17 (27). Research indicates that Th17/Treg cell imbalance plays a significant role in inflammatory responses, autoimmune diseases, and tumors. This imbalance may contribute to proteinuria generation, correlates closely with the severity of IgAVN renal injury, and influences the long-term prognosis of IgAVN.

### 3.3 Tfh cells

Follicular helper T cells (Tfh) are a CD4^+^ T cell subset recently identified as playing a primary role in assisting B cells. Tfh cells preferentially localize to CXCL13-rich B cell follicular zones via their characteristic surface molecule CXCR5. Within this niche, they sustain B cell germinal center responses by expressing CD40L, IL-4, and/or IL-21. Tfh cells play a critical role in regulating the initial differentiation of B cells into Ig-secreting plasma cells, Ig affinity maturation, and class switching. Tfh cells may be important in inducing B cell differentiation into plasma cells secreting polymeric IgA (pIgA) in children with IgAVN. Flow cytometric analysis of Tfh cell subsets revealed significantly higher numbers of CD4^+^CXCR5^+^, CD4^+^CXCR5^+^ICOS^+^, and CD4^+^CXCR5^+^PD-1^+^ Tfh cells in the peripheral blood of IgAVN patients compared to healthy controls (28). Specifically, CD4^+^CXCR5^+^Tfh cell counts showed a negative correlation with glomerular filtration rate (GFR), while CD4^+^CXCR5^+^ICOS^+^Tfh cell counts demonstrated a positive correlation with serum IgA levels and 24-hour urine protein quantification. Moreover, frequency of CD4^+^CXCR5^+^CCR6^+^Tfh17 cells were increased in IgAV patients and associated with disease severity. A significant infiltration of Tfh17 cells was found in the kidney of human IgAV nephritis patients (29). These findings suggest that these Tfh cell subsets may contribute to the pathogenesis and progression of IgAVN.

### 3.4 Regulatory B cells

Regulatory B cells (Bregs) are key regulators of immune responses in cancer, autoimmune diseases, and chronic viral infections. Experiments demonstrate that IgA-negative patients exhibit significantly lower Breg cell counts compared to healthy controls. Breg cells express regulatory cytokines IL-10 and transforming growth factor-β (TGF-β), suppressing IFN-γ and TNF-α secretion by effector T cells, promoting Foxp3+ Treg cell differentiation, and inhibiting dendritic cell (DC) function (28). IL-10 is a multifunctional regulatory cytokine that suppresses Th1- and Th2-like cytokine responses, various immune effector mechanisms, and the expression of molecules associated with inflammation or autoimmune diseases. It also downregulates IgE receptor and its signaling molecule expression while inducing the gene expression of terminator antibody IgG4 (30). Furthermore, IL-10 regulates Th17/Treg immune balance and participates in the pathogenesis of IgAV, particularly IgAVN.

### 3.5 Natural killer cells

Dysfunction of natural killer cells plays a significant role in the pathogenesis of IgAVN. Studies indicate that patients with IgAV and IgAVN exhibit a markedly reduced proportion of NK cells in their peripheral blood. This quantitative decrease is accompanied by severe functional impairment, which is more pronounced in IgAVN patients (31). In IgAV patients, activated NK cells failed to significantly upregulate glycolytic rates from resting levels, likely due to inhibition of the mTORC1 signaling pathway. This reduced glycolysis directly caused NK cell dysfunction, suggesting that abnormal metabolic reprogramming may be a key component in the pathogenesis of IgAV, potentially indirectly promoting persistent immune complex deposition in the kidneys and progression of inflammatory responses (32).

### 3.6 Macrophages

Macrophages serve as central executors of the innate immune response in the kidney, playing complex and critical roles in renal tissue injury and repair during IgAVN. During the pathophysiology of IgAVN, macrophages infiltrating the kidney primarily originate from circulating monocytes. Recruited to the glomeruli and renal interstitium by chemokines, they predominantly polarize into pro-inflammatory M1 and reparative M2 types. During disease activity, pro-inflammatory signals dominate, driving macrophage polarization toward the M1 phenotype. M1 macrophages recognize and phagocytose IgA immune complexes deposited in the glomerular mesangium and basement membrane via Fc receptors, a critical step in clearing pathogenic substances. However, while phagocytosing immune complexes, M1 macrophages undergo potent activation, releasing large amounts of proinflammatory cytokines such as tumor necrosis factor-α and interleukin-1β, and producing reactive oxygen species and other mediators (33). These inflammatory factors and mediators directly damage glomerular endothelial cells and mesangial cells, exacerbating local inflammation and directly causing proteinuria, hematuria, and declining renal function. As the disease becomes chronic or enters the repair phase, macrophage polarization may shift toward the M2 type. M2 macrophages participate in tissue repair and extracellular matrix remodeling by secreting anti-inflammatory factors and growth factors.

## 4 Inflammatory cytokines

Inflammatory cytokines act as proximate mediators that translate humoral and cellular immune dysregulation into tissue-level injury in IgAVN. TNF-α has been implicated in endothelial activation, coagulation disturbances and EMT via Smurf2/Akt signaling. IL-6 promotes activated B cells to secrete IgA and IgE and has been linked to IgA deposition in experimental studies. IL-16 and IL-18 levels correlate with renal damage severity and may serve as early indicators of IgAVN. IL-17 and IL-21 (produced by Th17/Tfh) intensify pro-inflammatory signaling via NF-κB and MAPK pathways, while IL-29 and IL-33 modulate Th1/Th2/Th17 polarization and dendritic cell maturation, thereby linking innate sensing to adaptive responses. This cytokine network therefore bridges immune complex deposition and cellular effectors to histopathological outcomes such as mesangial proliferation, crescent formation and fibrosis.

### 4.1 Interleukin-6, 10 (IL-6, 10)

IL-6 is a T cell- and macrophage-derived protein with pro-inflammatory and anti-inflammatory properties. It can play an important role in inducing inflammation by increasing the synthesis of acute-phase proteins and neutrophils in the bone marrow, while also inhibiting inflammation by suppressing TNF-α and IL-1 and activating IL-10 (30). Reports show that the expression of IL-6 in the serum of patients with purpuric nephritis was significantly higher than in the normal control group and the allergic purpura group. IL-6 can induce activated B cells to secrete IgA and IgE, promote the formation of circulating immune complexes and their deposition in blood vessels and glomerular mesangial areas, promote the proliferation of glomerular mesangial cells, leading to the formation of glomerulonephritis (34).

### 4.2 Interleukin-16, 18 (IL-16, 18)

IL-16 is an immunomodulatory chemokine that can be produced in various cells, while IL-18 is a pro-inflammatory cytokine that plays an important role in both adaptive and innate immunity. Serum IL-16 and IL-18 levels in children with IgAVN were elevated and showed an upward trend with the aggravation of renal damage, which can serve as reference indicators for predicting early IgAVN (35). IL-16 can chemotactically attract T cells and monocytes to aggregate in blood vessels, triggering inflammatory responses, and upregulate the expression of inflammatory factors such as IL-6 and γ-interferon, thereby participating in the occurrence and development of vasculitis. In addition, IL-16 can stimulate B cells to secrete IgA, form IgA immune complexes, deposit on capillary walls to cause inflammation, and IgA immune complexes deposited in the glomerular mesangial area will cause mesangial proliferation, leading to renal damage. Reports indicate that IL-18 has a fibrotic effect, and studies on ureteral obstruction models and aldosterone/salt models have proven a direct association between IL-18 levels and the severity of unilateral renal fibrosis. Experimental data show that IL-18 can upregulate the expression of fibronectin, type I collagen, and α-SMA in mouse kidneys and exacerbate renal fibrosis. IL-18 also plays an important role in inducing the development of renal fibrosis by regulating the infiltration of inflammatory cells, the expression of inflammatory cytokines and chemokines, and the transformation of bone marrow-derived M2 macrophages into fibroblasts (36).

### 4.3 Interleukin-17, 21 (IL-17, 21)

Both IL-17 and IL-21 can be produced by Th17, where IL-17 is the signature cytokine produced by Th17 cells, and IL-21 can indirectly regulate IL-17 levels to participate in immune responses. Numerous studies show that IL-17 and IL-21 levels in children with IgAVN are significantly higher than in healthy children. IL-17 can upregulate the expression of pro-inflammatory genes by activating nuclear transcription factor κB (NF-κB), MAP kinase (MAPK), and C/EBP cascade reactions, thereby amplifying inflammatory responses, causing damage to renal vascular endothelial cells, exacerbating renal ischemia, and ultimately leading to glomerular and tubular damage (37). IL-21 can regulate the production and polarization of T cells and B cells through its receptor IL-21R via JAK/STAT, MAPK, and PI3K pathways, and affect the functions of Th cells, plasma cells, and germinal center cells. IL-21 and IL-6 can induce Tfh cell differentiation, while IL-21 deficiency leads to reduced Tfh polarization and decreased Bcl-6 expression. IL-21 participates in immune responses by indirectly inducing Th17 cell proliferation, regulating their function, and regulating IL-17 production by Th17 cells. IL-21 enhances the resistance of CD4+ T cells to Treg cell suppression by reducing IL-2 production in T cells in a STAT3- and STAT5-dependent manner, indirectly reducing the absolute number and differentiation of Treg cells. Excessive IL-21 enhances the tolerance of CD4+ T cells to Treg cells by inhibiting the production and development of Treg cells, thereby blocking the role of Treg cells in immune tolerance and protection (38).

### 4.4 Interleukin-29 (IL-29)

IL-29 is a multifunctional inflammatory cytokine that can trigger and amplify inflammatory responses, belonging to the interferon IFN-λ family, with various immunomodulatory activities such as antiviral, antiproliferative, and antitumor properties. It participates in regulating T helper cell Th1/Th2 responses and modulating the functions of macrophages, B cells, and plasmacytoid dendritic cells. IL-29 plays an important role in the pathogenesis of HSP, possibly through increased IL-29 levels activating the NF-κB signaling pathway, further inducing excessive Th2 activation and cytokine overrelease, and in severe cases triggering HSP (39). IL-29 mRNA levels in peripheral blood mononuclear cells of children with acute HSP were significantly higher than in the control group, suggesting a close relationship between IL-29 and the pathogenesis of HSP, and also indicating that IL-29 in HSP patients has changed at the transcriptional level (39).

### 4.5 Interleukin-33 (IL-33)

IL-33 is highly secreted in various autoimmune diseases and is expressed in intestinal endothelial cells, epithelial cells, and fibroblasts. Researchers have observed elevated IL-33 expression in the serum of children with IgAVN, which is related to pathological types; the higher the pathological grade, the higher the IL-33 concentration in the serum. IL-33 can induce DC maturation, and IL-33-matured DCs (IL-33-matDCs) can inhibit the differentiation of CD4+ T cells into Treg cells. Experiments have shown that under Treg polarization conditions, IL-33-matDCs interrupt Treg differentiation in an IL-33 dose-dependent manner. IL-33 triggers DCs to secrete IL-1β and IL-6, as well as DC maturation, thereby inducing Th17 cell differentiation from naive CD4^+^T cells. IL-33-matDCs can also convert stable Treg cells into Th17 cells through IL-6 signaling, while having almost no effect on unstable Treg cells (27). IL-33 can stimulate mast cells, induce degranulation, promote cell maturation, and cause IL-1 and other co-proteins to bind to ST2, activating myeloid differentiation factor, and regulating the formation of various inflammatory factors in the body through transcriptional effects (40) (**Table 1**).

**Table 1:** Different cytokines their functions and key findings in IgAVN.

Cytokine	Main functions	Key findings in IgAVN
IL-6, IL-10	① Pro- and anti-inflammatory properties ② Induces acute-phase proteins and neutrophils ③ Suppresses TNF-α and IL-1, activates IL-10	① Significantly elevated in serum of purpuric nephritis patients vs. healthy controls and allergic purpura group ② Induces B cells to secrete IgA and IgE ③ Promotes circulating immune complex formation and deposition in vessels and glomerular mesangium ④ Causes mesangial cell proliferation and glomerulonephritis
IL-16, IL-18	① IL-16: immunomodulatory chemokine, attracts T cells and monocytes ② IL-18: pro-inflammatory cytokine involved in innate and adaptive immunity, promotes fibrosis	① Both elevated in children with IgAVN; levels increase with severity of renal damage ② IL-16 upregulates IL-6 and IFN-γ, stimulates IgA secretion and immune complex deposition ③ IL-18 associated with renal fibrosis ④ Regulates inflammatory cell infiltration and M2 macrophage-to-fibroblast transition
IL-17, IL-21	① Both produced by Th17 cells ② IL-17: signature Th17 cytokine ③ IL-21: regulates IL-17 production and T/B cell function	① Significantly higher in children with IgAVN than healthy controls ② IL-17 activates NF-κB, MAPK, C/EBP → amplifies inflammation, damages renal vascular endothelium, causes glomerular and tubular injury ③ IL-21 acts via JAK/STAT, MAPK, PI3K pathways ④ Promotes Tfh differentiation (with IL-6), inhibits Treg development and function, indirectly enhances Th17 activity
IL-29	① Member of IFN-λ family ② Triggers and amplifies inflammatory responses ③ Regulates Th1/Th2 balance and functions of macrophages, B cells, pDCs	① Elevated in HSP; activates NF-κB pathway → excessive Th2 activation and cytokine release ② IL-29 mRNA levels in PBMCs significantly higher in acute HSP children vs. Controls ③ Closely associated with HSP pathogenesis at transcriptional level
IL-33	① Alarmin cytokine highly expressed in autoimmune diseases ② Expressed in endothelial, epithelial cells and fibroblasts	① Serum levels elevated in IgAVN children; positively correlated with pathological grade ② Induces DC maturation and inhibits Treg differentiation, promotes Th17 differentiation (via IL-1β, IL-6) ③ Converts stable Treg cells into Th17 cells through IL-6 signaling ④ Stimulates mast cell degranulation and inflammatory factor production

### 4.6 Tumor necrosis factor-α

TNF-α is a multifunctional inflammatory cytokine that has immune defense functions and can also serve as an important mediator in the occurrence of inflammation, injury, and even shock in the body. Studies have found that serum TNF-α levels in children with IgAVN were higher than in children with HSP (41). In *in vitro* study, TNF-α can significantly upregulate Smurf2 in proximal tubular cells, and overexpression of Smurf2 can promote the expression of α-smooth muscle actin (α-SMA) and inhibit the expression of E-cadherin (E-cad). In addition, TNF-α-induced Smurf2 expression promotes EMT through the Akt signaling pathway and may promote the pathogenesis of renal interstitial fibrosis (42). TNF-α also plays an important role in endothelial cell activation, causing coagulation and microcirculatory disorders, thereby leading to necrosis of vascular endothelial cells. TNF-α can also induce vascular endothelial cells to produce TGF-β, where high concentrations of TGF-β1 promote the proliferation of renal mesangial cells, induce deposition of extracellular matrix, promote glomerular sclerosis, and induce fibroblast proliferation, leading to renal tissue fibrosis (43). Clinical studies have also confirmed that TGF-β1 can participate in a series of renal pathological changes such as crescent formation, vascular proliferation, and immune complex deposition, exacerbating the deterioration of kidney disease.

### 4.7 Transforming growth factor-β

TGF-β plays a complex and contradictory role in the pathological process of IgAVN. On one hand, TGF-β is a crucial immunoregulatory factor with anti-inflammatory and immunosuppressive functions, protecting the kidneys from damage caused by excessive immune responses. On the other hand, within the chronic inflammatory environment of IgAVN, the pro-fibrotic effects of TGF-β often dominate, becoming a core factor driving progressive renal sclerosis and functional loss. TGF-β is also a key driver of tubulointerstitial fibrosis. It induces EMT in tubular epithelial cells, transforming them into myofibroblasts with matrix-secreting capabilities, and stimulates fibroblast activation. Together, these processes lead to collagen deposition and fibrosis in the renal interstitium (44). Thus, TGF-β exhibits a “double-edged sword” characteristic in IgAVN, and targeted modulation of the TGF-β signaling pathway may represent a novel strategy to intervene in IgAVN progression.

### 4.8 Interferon

Studies indicate a significant deficiency in the interferon signaling pathway within peripheral blood mononuclear cells of IgAVN patients, accompanied by reduced IFN-γ secretion. This leads to impaired cytotoxic and immunoregulatory functions of natural killer (NK) cells (45). IFN-γ promotes M1 polarization of macrophages, enhancing their antigen-presenting capacity and secretion of proinflammatory factors such as TNF-α and IL-6. IFN-γ upregulates HLA-DR expression in glomerular mesangial cells, promoting local immune complex deposition (46). Clinical studies reveal a positive correlation between IFN-γ levels in renal tissue and the intensity of glomerular C3 deposition in IgAN patients, with severe C3 deposition often associated with poorer long-term renal prognosis (47).

### 4.9 Chemokines

**Figure 2:**
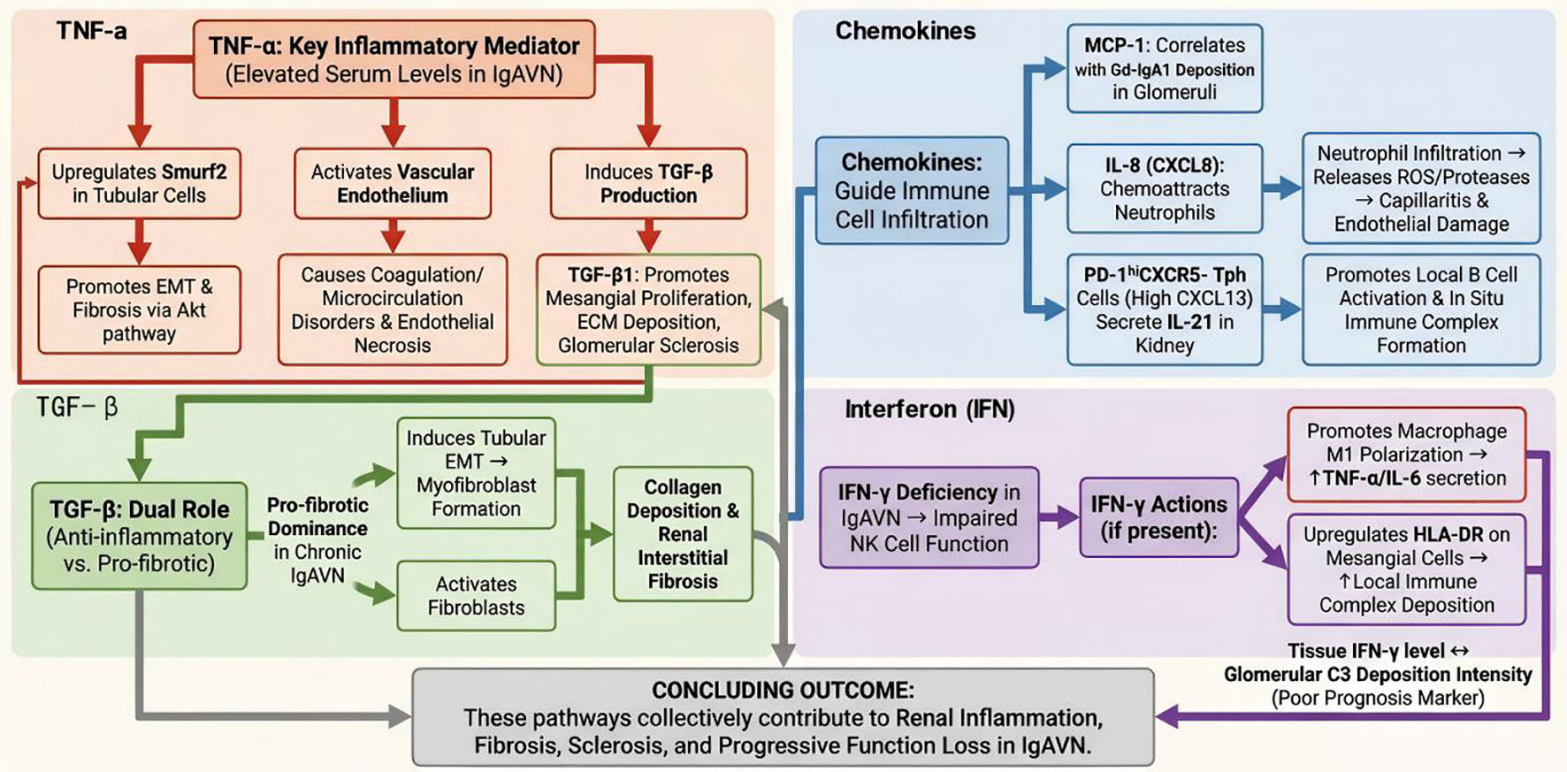
Role of tumor necrosis factor-a (TNF-a), chemokines, and growth factors.

Chemokines establish chemical gradients to precisely guide different immune cell subsets toward inflammatory sites, playing a crucial role in renal immune cell infiltration and local inflammatory amplification in IgAVN. In renal tissues of IgAVN patients, MCP-1 levels positively correlate with the deposition intensity of Gd-IgA1 within glomeruli, suggesting its involvement in IgA immune complex-mediated inflammation. IL-8/CXCL8 primarily chemotaxes neutrophils toward the kidneys. Neutrophils, as early effector cells in acute inflammation, release reactive oxygen species and proteases upon infiltration, directly damaging glomerular capillary endothelium—a process closely linked to the characteristic “capillaritis” pathology of IgAVN (48). Studies reveal increased frequencies of PD-1^hi^CXCR5^-^ CD4^+^ T peripheral helper cells (Tph) with high CXCL13 expression in the peripheral blood of IgAVN patients. These Tph cells induce B-cell differentiation and antibody production locally in the kidney by secreting cytokines such as IL-21, potentially promoting the formation of *in situ* immune complexes and chronic renal inflammation (29) (**Figure 2**).

## 5 Integrated mechanistic synthesis and implications

**Figure 3:**
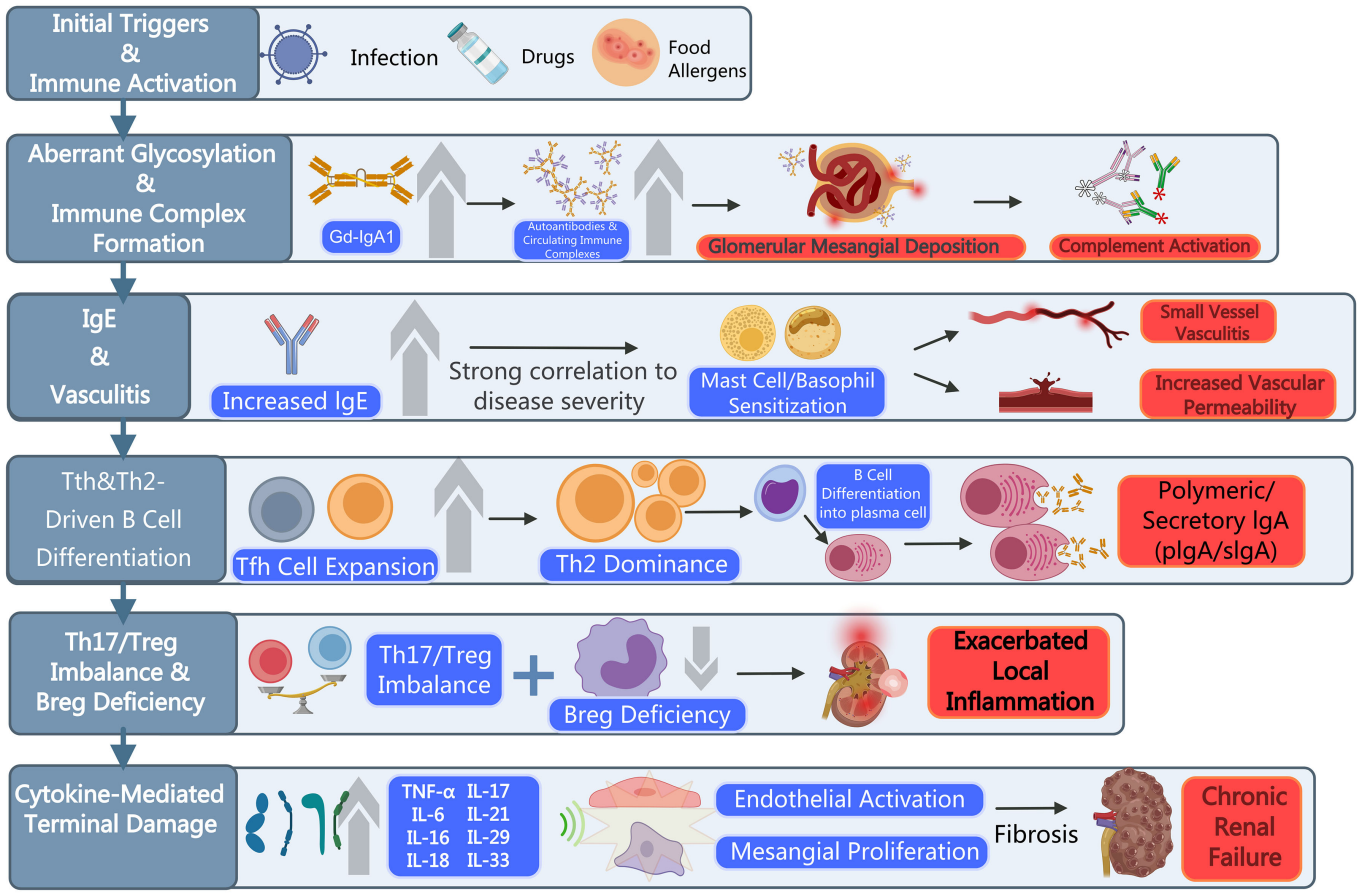
Integrated mechanistic framework for IgAVN. Key nodes and interactions synthesized: (1) initiating triggers (infections, drugs, food allergens) provoke mucosal and systemic immune activation; (2) aberrant O-glycosylation of IgA1 generates Gd-IgA1, which elicits anti-Gd-IgA1 autoantibodies and circulating immune complexes that deposit in the glomerular mesangium and activate complement; (3) elevated IgE may sensitize mast cells/basophils and promote small-vessel vasculitis, increasing vascular permeability; (4) Tfh expansion and Th2 predominance favor pathological B cell differentiation toward polymeric/secretory IgA production; (5) Th17/Treg imbalance and Breg deficiency amplify local inflammation and reduce immunoregulatory control; (6) cytokines (TNF-α, IL-6, IL-16, IL-18, IL-17, IL-21, IL-29, IL-33) mediate endothelial activation, mesangial proliferation, EMT and fibrogenesis, ultimately leading to crescent formation, glomerulosclerosis and progression to chronic renal failure.

Based on the evidence summarized above, we propose the following integrated mechanistic model for IgAVN (**Figure 3**):

Initiation: Exogenous triggers (infections, drugs, food allergens) provoke mucosal and systemic immune activation, which in susceptible individuals results in aberrant O-glycosylation of IgA1 and increased circulating galactose-deficient IgA1 (Gd-IgA1).

Immune complex formation and deposition: Gd-IgA1 triggers production of anti-Gd-IgA1 autoantibodies (IgG/IgA). Circulating immune complexes formed thereby deposit in the glomerular mesangium and small-vessel endothelium and activate complement pathways, leading to mesangial cell activation, macrophage/lymphocyte recruitment and local inflammation.

Humoral-cellular amplification loops: Expansion of Tfh cells and Th2 predominance favor pathological B cell differentiation toward polymeric IgA-secreting plasma cells and increased IgA/IgE production. Th17/Treg imbalance and Breg deficiency further reduce regulatory control and sustain pro-inflammatory cytokine production.

Cytokine-mediated tissue injury and fibrogenesis: Cytokines including TNF-α, IL-6, IL-16, IL-18, IL-17, IL-21, IL-29 and IL-33 drive endothelial activation, NF-κB/MAPK signaling, EMT, extracellular matrix deposition and profibrotic responses, ultimately producing crescent formation, glomerulosclerosis and progressive renal fibrosis.

## 6 Brief perspectives on therapeutic research

Advances in the understanding of IgAVN immunopathogenesis may provide a basis for future biomarker development and mechanism-based therapeutic exploration. In particular, key processes such as aberrant Gd-IgA1 production, immune complex deposition, complement activation, T-cell subset imbalance, B-cell dysregulation, and cytokine network activation may represent potential points for translational investigation. However, current evidence remains limited, and most findings require further validation before clinical application. Future studies should continue to clarify how these immune mechanisms contribute to disease heterogeneity, renal injury severity, and therapeutic responsiveness in IgAVN.

## 7 Conclusion

In summary, IgAVN pathogenesis is driven by an intricate network involving aberrant humoral immunity (Gd-IgA1 production and complement activation), dysregulated cellular immunity (Th1/Th2 and Th17/Treg imbalances), and a complex cytokine cascade. A deeper understanding of these synergistic immune mechanisms is essential for guiding the development of targeted immunotherapies and improving the prognosis of IgAVN patients.
